# Use of Chitosan from Southern King Crab to Develop Films Functionalized with RGD Peptides for Potential Tissue Engineering Applications

**DOI:** 10.3390/biomimetics8030323

**Published:** 2023-07-21

**Authors:** Juan Carlos Forero, Karina Carvajal, Fanny Guzmán, Cristian Acevedo, Nelson Osses, Paula Santana

**Affiliations:** 1 Escuela de Ciencias de la Salud, Universidad de Viña del Mar, Viña del Mar 2580022, Chile; juan.forero@uvm.cl; 2 Centro Interdisciplinario de Neurociencia de Valparaíso, Facultad de Ciencias, Universidad de Valparaíso, Valparaíso 2360102, Chile; karina.carvajal@postgrado.uv.cl; 3 Núcleo Biotecnología Curauma, Pontificia Universidad Católica de Valparaíso, Valparaíso 2340025, Chile; fanny.guzman@pucv.cl; 4 Departamento de Física, Universidad Técnica Federico Santa María, Valparaíso 2390123, Chile; cristian.acevedo@usm.cl; 5 Instituto de Química, Facultad de Ciencias, Pontificia Universidad Católica de Valparaíso, Valparaíso 2340025, Chile; 6 IMPACT, Center of Interventional Medicine for Precision and Advanced Cellular Therapy, Santiago 7620086, Chile; 7 Instituto de Ciencias Aplicadas, Facultad de Ingeniería, Universidad Autónoma de Chile, Santiago 8910060, Chile

**Keywords:** crab shell waste, chitosan, films, RGD peptides, tissue engineering, cell adhesion

## Abstract

Southern King Crab (SKC) represents an important fishery resource that has the potential to be a natural source of chitosan (CS) production. In tissue engineering, CS is very useful to generate biomaterials. However, CS has a lack of signaling molecules that facilitate cell–substrate interaction. Therefore, RGD (arginine–glycine–aspartic acid) peptides corresponding to the main integrin recognition site in extracellular matrix proteins have been used to improve the CS surface. The aim of this study was to evaluate in vitro cell adhesion and proliferation of CS films synthesized from SKC shell wastes functionalized with RGD peptides. The FTIR spectrum of CS isolated from SKC shells (SKC-CS) was comparable to commercial CS. Thermal properties of films showed similar endothermic peaks at 53.4 and 53.0 °C in commercial CS and SKC-CS, respectively. The purification and molecular masses of the synthesized RGD peptides were confirmed using HPLC and ESI-MS mass spectrometry, respectively. Mouse embryonic fibroblast cells showed higher adhesion on SKC-CS (1% *w*/*v*) film when it was functionalized with linear RGD peptides. In contrast, a cyclic RGD peptide showed similar adhesion to control peptide (RDG), but the highest cell proliferation was after 48 h of culture. This study shows that functionalization of SKC-CS films with linear or cyclic RGD peptides are useful to improve effects on cell adhesion or cell proliferation. Furthermore, our work contributes to knowledge of a new source of CS to synthesize constructs for tissue engineering applications.

## 1. Introduction

In recent years, development of functional and innovative scaffolds for tissue regeneration has brought the attention of researchers and biotech companies focused on regenerative medicine. In this sense, biomaterials have been used to fabricate different polymeric scaffolds to be used as implants to replace or repair damaged tissues [1]. One of the most widely used biomaterials in tissue engineering is chitosan (CS) due to its biocompatibility, biodegradability, and bacteriostatic effect [2]. CS can be isolated from different natural sources such as crustaceans, fungi, and insects. However, waste from crustaceans (crabs and crayfish) is the main source of obtention due to the large amount of the crustacean exoskeleton available as a byproduct of food processing [3]. In this context, Southern King Crab (*Lithodes centolla*; SKC hereafter) represents an important fishery resource in South America and southern Chile [4], and waste from this commercial species provides a potential natural source of CS production at an industrial scale. CS has been a useful biomaterial to fabricate constructs such as film, sponge, membrane, fiber, and hydrogels for tissue engineering applications due to the presence of free amine groups in its backbone chains, which bring additional functional properties after chemical modifications [5]. However, the main disadvantage of CS for tissue engineering and regenerative medicine is its poor cell–matrix interaction due to the lack of bioactive cell signaling molecules [6]. Therefore, modifying the surface of CS with bioactive molecules is a useful strategy to improve cell adhesion, proliferation, or differentiation. In addition, CS has been shown to be a versatile material to fuse biomimetics and regenerative medicine that is referred to as “tissue bionics” [7]. By integrating the principles of bionics with the field of tissue engineering, researchers can further enhance the development of biomaterials and scaffolds [7]. These bionic-inspired biomaterials, such as chitosan films functionalized with bioactive molecules, exhibit improved properties that mimic the natural extracellular matrix [8]. They provide an optimal microenvironment for cell adhesion, proliferation, and differentiation, promoting bionic-like tissue scaffolds that closely mimic the native and have been used for bone [9], skin [10], vascular [11] and nerve [12] regeneration.

Among different CS surface modifications, peptides derived from extracellular matrix (ECM) proteins have been generally probed [13]. Some studies have used specific motifs of ECM proteins to drive a desired cellular response [9,14]. However, regardless of CS’s potential application in tissue engineering, functionalization with arginyl–glycyl–aspartic acid (RGD) peptides and RGD derivatives are the most used bioactive motif [13]. RGD is a tripeptide sequence present in different ECM proteins that is recognized by cells through integrins—membrane receptors enhancing cell adhesion proliferation, or differentiation [15,16,17]. Synthesized RGD peptides avoid the use of ECM proteins and can be used in a controlled way to obtain a biomimetic material. For instances, densities between 6×105 and 6 × 10^11^ RGD ligands/mm^2^ are sufficient for adhesion, spreading and formation of focal adhesions [18]. Functionalization of polymeric scaffolds with peptides can be performed using various methods, such as inclusion in the bulk material, using a carrier, by adsorption or by covalent linking to the biomaterial surface. In this sense, covalent immobilization of RGD peptides on CS surfaces using N-hydroxysuccinimide (NHS) and 1-ethyl-3-(3-dimethylaminopropyl) carbodiimide (EDC) offers several advantages: (a) enhanced stability, which allows for long-term use and repeated exposure to various environmental conditions without compromising the integrity and activity of the immobilized peptides [19,20]; (b) retention of peptide bioactivity. NHS and EDC immobilization methods typically involve mild reaction conditions, which helps preserve the bioactivity of the peptides. These reagents do not require harsh temperatures or solvents that could denature or degrade the peptides [21]; and (c) control over peptide density and orientation. The NHS and EDC method offers control over the density and orientation of immobilized peptides on the CS surface by adjusting concentrations of NHS, EDC, and peptides, as well as the reaction time [22]. It is important to highlight that surfaces of biomaterials are the first structure that comes into contact with the biological environment [23]. Therefore, modifying the surface properties of these materials, such as CS, provides initial biomimetic support to cells so that they can subsequently produce their own ECM.

Although some studies have shown that the combination of CS and RGD have a potential application in developing new promising scaffolds for tissue engineering [24], to date, there are no studies evaluating the use of CS from Southern King Crab (SKC-CS) for these purposes. Therefore, we hypothesize that surface functionalization of CS films derived from SKC waste with RGD peptides could improve in vitro cell behavior. Thus, the aim of this study was to obtain SKC-CS films functionalized with RGD peptides and evaluate cell adhesion and viability of Mouse Embryonic Fibroblasts (MEFs) to demonstrate the feasibility of CS isolated from this natural source for tissue engineering applications.

## 2. Materials and Methods

### 2.1. Sample Collection

Crab shell waste was collected from marine food processing companies in Calbuco, southern Chile. The crab exoskeleton waste was placed in ziploc bags and dried under room temperature for 10 days and ground well to make a powder.

### 2.2. Materials

Potassium hydroxide (KOH), sodium hydroxide (NaOH) and chlorhydric acid (HCl) were purchased from Winkler Ltd.a. (Santiago, Chile). Commercial CS and Cell proliferation reagent WST-1 were purchased from Merck/Sigma-Aldrich (Darmstadt, Germany), Dulbecco’s modified Eagle’s medium (DMEM) was acquired from Hyclone (Chicago, IL, USA). Fetal bovine serum (FBS) was purchased from GIBCO (Waltham, MA, USA). All other reagents and solvents were obtained from commercial suppliers. All aqueous solutions were prepared with ultrapure water (<18.2 MΩ-cm) from PURELAB classic ELGA Milli-Q system (Paris, France).

### 2.3. Isolation and Characterization of CS

**Figure 1:**
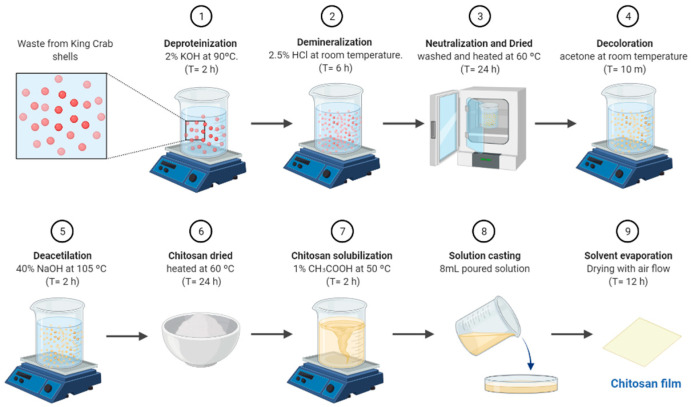
Schematic procedure for isolation of chitosan from Southern King Crab and synthesis of SKC-CS films. (1–5) Illustration of chemical procedure of chitosan production. (6–9) Synthesis of chitosan films by solvent casting.

CS from SKC shells was isolated using the methods previously reported by Sarbon et al. 2014 [25]. Briefly, 2% KOH and 2.5% HCl aqueous solutions were used for deproteinization and demineralization, respectively. The deacetylation of the resulting chitin was performed using 40% NaOH at 105 °C for 2 h. The detailed procedure is shown in Figure 1. CS characterization was performed according to analytical methods previously published [26]. For spectra analysis an WQF-520 FT-IR spectrometer (Beijing-China) was used. The degree of deacetylation (*DDA*) was calculated by using equation.

(1) $$DDA\left[\%\right]=\frac{2.03(v2-v1)}{m+0.0042\;(v2-v1)}$$

where, *m* is the weight of the sample, 2.03 is the coefficient resulting from the molecular weight of chitin monomer unit and 0.0042 is the coefficient resulting from the difference between molecular weights of chitin and chitosan monomer units. *v*1 and *v*2 are the two inflection points corresponding to neutralization of HCl and neutralization of the ammonium ions from CS [27].

### 2.4. Synthesis of SKC-CS Films

SKC-CS films were made by cold casting using 1%, 2% and 3% CS solution (*w*/*v*). The final solution was poured into petri dishes, setting the volume to obtain 5 mm of height, then they were kept for 3 days at 10 °C [28]. 

### 2.5. Thermal Properties of SKC-CS Films

The thermal properties were analyzed by using a differential scanning calorimeter (DSC1 STARe System, METTLER-TOLEDO, Switzerland). A total of 10 mg of CS film was hermetically sealed in a 100 μL stainless-steel pan. The thermal scanning conditions were as follows: heating from −20 to 120 °C at 10 °C/min, holding at 120 °C for 1 min, cooling from 120 to −20 °C at 20 °C/min, holding at −20 °C for 2 min, and reheating to 120 °C at 10 °C/min. Tm and *Tg* were determined as the onset of the endothermic peak [29] and the midpoint of the change in heat capacity (*Cp*) observed in the first heating scan [30], respectively.

### 2.6. Measurement of Swelling Percentage

Stability analyses were conducted to examine the durability of the chitosan film. This was accomplished by observing the change in mass of the chitosan films after they reached a state of equilibrium by swelling in PBS (pH 7.4, 1×) at a temperature of 37 °C. CS films were weighted, which served as the initial mass (ms) and subsequently submerged, and the mass of the swollen chitosan film was recorded after 100 min. The mass of the swollen sample at a specific time point denoted as mt, was also measured. The ratio of mt/ms × 100 indicates the weight alteration and provides insights into the stability of the chitosan film [31].

### 2.7. Synthesis and Characterization of RGD Peptides

The linear RGD peptides GRGD, RGDG and RDG (negative control peptide), as well as the cyclic RGD peptide (cRGDK), were synthesized using the Fmoc solid phase peptide synthesis (Fmoc-SPPS) strategy on Rink amide resin (Iris) (0.59 mmol/g substitution) as has been described previously [32,33]. The couplings were performed using 3 equivalents, relative to the resin substitution, of amino acid, activator, and OxymaPure^®^, along with 4.5 equivalents of N-ethyldiisopropylamine (DIPEA) (3/3/3/4.5). The couplings were monitored using the ninhydrin test [34].

For the synthesis of the cRGDK cyclic peptide, the first amino acid used was Fmoc-Lys(Dde)-OH and HBTU (N-[(1H-benzotriazol-1-yl)-(dimethylamino)methylene]-N-methylmethanaminium hex-afluorophosphate N-oxide) was used as an activating reagent for the first coupling. While, for second and third coupling TBTU (N-[(1H-benzotriazol-1-yl)(dimethylamino)-methylene]-N-methylmethanaminium tetra-fluoroborate N-oxide) and HCTU (N-[6-chloro(1H-benzotriazol-1-yl)-(dimethylamino)methylene]-N-methylmethanaminium hexafluorophosphate N-oxide) were used, respectively, in dimethylformamide (DMF). The following three amino acids (RGD) were incorporated as Fmoc-amino acids with the same activation chemistry.

For the cyclization, the Dde side-chain protection group of lysine was removed by treatment with 2% hydrazine monohydrate in DMF (*v*/*v*) on resin. Then, malonic acid was coupled using the same coupling strategy as for a regular amino acid. Subsequently, the Fmoc group of the terminal arginine was deprotected, and the peptide bond was formed with the second carboxyl group of malonic acid using TBTU as the activator.

Finally, the peptides were cleaved from the resin using a mixture of trifluoroacetic acid/ultrapure water/triisopropylsilane/ethanedithiol (92.5/2.5/2.5/2.5) (*v*/*v*/*v*/*v*) for 120 min at room temperature. The peptides were precipitated with cold diethyl ether, dried, solubilized with Milli-Q water, and then lyophilized. The purity of the peptides was verified by RP-HPLC using a JASCO system (JASCO Corp., Tokyo, Japan) with a Water Corp XBridge™ BEH C18 column (100 × 4.6 mm, 3.5 μm) using a gradient of 0–70% acetonitrile in water containing 0.05% TFA as solvent A and acetonitrile containing 0.05% TFA as solvent B, at a flow rate of 1 mL/min for 8 min. The peptides were characterized by mass spectrometry (MS) using an LCMS-2020 ESI–MS instrument (Shimadzu Corp., Kyoto, Japan). The peptides were purified using C18 extraction columns (Clean-Up^®^, United Chemical Technologies 200 mg 3 mL, Bristol PA, USA) with an increasing gradient of acetonitrile/water, then frozen and lyophilized.

### 2.8. Functionalization of SKC-CS Films with RGD Peptides

SKC-CS films were functionalized by immobilizing purified RGD peptides (GRGD, RGDG, cRGDK) on the CS surface [35]. As a negative control RDG peptide was used. Solutions of each peptide (0.2 mM) with EDC (1-Ethyl-3-[3-dimethylami-nopropyl]-carbodiimide hydrochloride; 13 mM) and NHS (N-hidroxisuccinimida; 5.3 mM) in PBS (phosphate buffered saline) were prepared at a 25:25:1 ratio, respectively. The films were immersed into the peptide solution at 4 °C for 72 h to assure that the immobilization reaction was complete. The conjugation of RGD onto SKC-CS film was analyzed by FTIR (Fourier transform infrared) spectroscopy. The spectra of samples were taken at 500–4500 cm^−1^ wavelength (FT-IR ATR Jasco 6000).

### 2.9. Cell Culture Studies

MEFs were cultured in 60 × 15 mm dishes (Corning, NY, USA) in DMEM with 10% fetal bovine serum containing l-glutamine and penicillin–streptomycin at 37 °C in air containing 5% CO_2_. CS films with a diameter of 1.9 cm^2^ were disinfected by immersion in ethanol 70% (*v*/*v*) overnight. Then, the polymer was washed and hydrated for 2 h with PBS prior to cell seeding; thereafter, scaffolds were placed in a 24-well cell culture plate. Then, 1 × 10^5^ or 2 × 10^4^ cells/cm^2^ were seeded for adhesion and cell proliferation, respectively. Attached cells after 4 h of seeding were visualized by Hoechst 33342 (5 µM) staining. From the micrograph, a quantification of the nuclear-to-cytoplasmic area was performed using Image J software, version 1.51k. Quantitative cell biomass was estimated by the resazurin assay. After incubation periods post-seeding (4, 24 and 48 h), cells were washed with DMEM and incubated with resazurin (4 µg/mL) (Merck/Sigma, Germany) for 4 h at 37 °C in DMEM with humidified air. Once resazurin was incubated, 100 μL was taken from each sample and the optical density (OD) was measured at 570 nm in an Appliskan™ (Thermo Fisher Scientific, Waltham, MA USA) [36]. For initial adhesion determination, cells were loaded over the CS–RGD films and left for 4 h in standard culture conditions. After that, CS–RGD films were removed, and the attached biomass was measured as indicated before at 570 nm and estimated by Equation (2):

(2) $$Cell\;adhesion\;(\%):\;\frac{{Mean\;OD}_{570f}}{Mean\;{OD}_{570fp}}\;\times \;100,$$

where *OD*_570*f*_ represents the measured absorbances for cells attached to the film and *OD*_570*fp*_ represents the sum of absorbance from cells attached to the film and cells remaining on the culture plate. For proliferation assays, cell biomass on films was determined using Equation (3):

(3) $$Cell\;viability\;\left(\%\right):\;\frac{{Mean\;OD}_{570f}}{{Mean\;OD}_{570i}}\;\times \;100,$$

where *OD*_570*f*_ represents the optical densities of the viable biomass on films with RGD peptides at 24 or 48 h, and *OD*_570*i*_ represents the optical density of the initial (4 h) amount of cell biomass attached to the films [37].

### 2.10. Statistical Analysis

Experiments were performed in triplicate unless indicated otherwise. The data are expressed as the mean ± standard deviation. Basic statistical analyses (*t*-test, ANOVA and two-way ANOVA) were performed by using statistical tools in the software GraphPad Prism version 4.0 (San Diego, CA, USA). Differences at the level of *p* < 0.05 were accepted as significant.

## 3. Results

### 3.1. Isolation and CS Characterization

To characterize SKC-CS, we evaluated parameters such as moisture content that determines the amount of water present, which is crucial for product stability and shelf life [38]. Ash content indicates the presence of inorganic impurities, which can affect the chitosan’s properties and performance [39]. On the other hand, the nitrogen content is directly related to the chitosan’s molecular weight and the degree of deacetylation that indicates the extent to which the polymer has been deacetylated, affecting its solubility, biocompatibility, and other properties [40]. Table 1 shows the comparison of these parameters for isolated SKC-CS and commercial CS.

**Figure 2:**
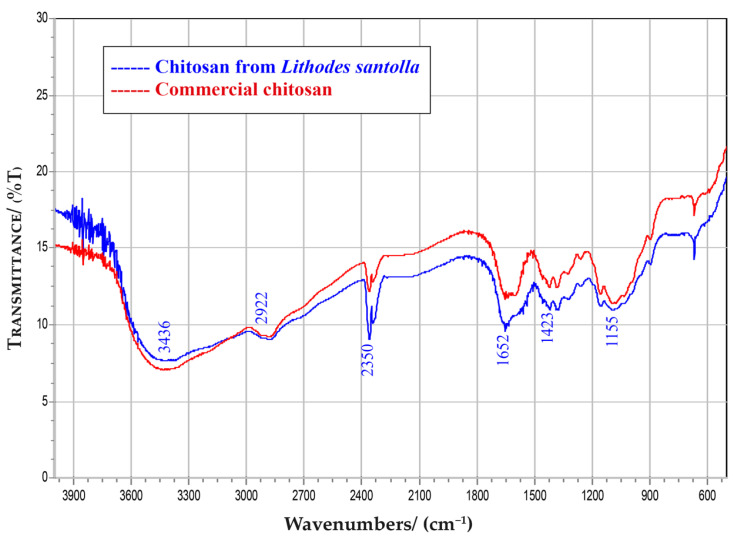
FTIR spectra for chitosan from SKC-CS and from a commercial sample. Plot generated shows similar spectra between samples.

Fourier transform infrared (FTIR) spectroscopy is the more conclusive evidence to determine the vibration of functional groups in chitin and CS [41]. Figure 2 shows the FTIR spectrum of isolated CS from SKC and commercial CS, which have similar shape and peaks. The peak observed at 3436 cm^−1^ corresponds to the stretching vibration of free hydroxyl and N–H bonds in amino group. The peak at 2922 cm^−1^ is indicative of aliphatic compounds, C-H stretch. The peaks at 2350 cm^−1^, correspond to the vibration of CO_2_. The peak 1652 cm^−1^ is attributed to the stretching of the C=O group. The peak 1423 cm^−1^ corresponds to the vibrational modes of the CH_2_ group and the peak 1155 cm^−1^ indicated the presence of ether bonds (C-O-C) [42,43,44].

### 3.2. DSC Analysis

**Figure 3:**
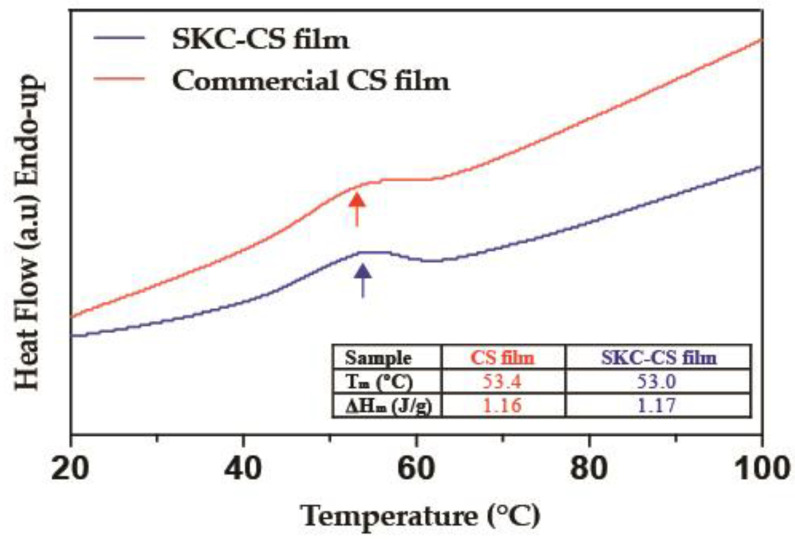
DSC thermogram from CS films. The red line shows the thermal properties of the SKC-CS film and the blue line represents the heat flow from commercial CS film, arrows show similar melting temperatures in both.

The thermal properties of SKC-CS films were investigated via DSC. Prior to the analysis, the films were equilibrated in a chamber with ≈75% relative humidity. Figure 3 shows endothermic peaks at 53.4 and 53.0 °C in films fabricated with commercial CS and SKC-CS, respectively. 

### 3.3. Characterization of RGD Peptides

**Figure 4:**
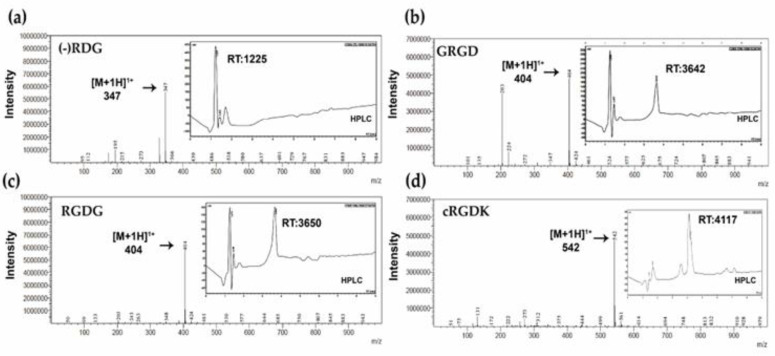
Characterization of synthetized RGD peptides. Plots show mass spectra and HPLC chromatograms of linear RGD peptides, (**a**) RDG (346.3 *m*/*z* theoretical), (**b**) GRGD (403.4 *m*/*z* theoretical), (**c**) RGDG (403.4 *m*/*z* theoretical) and cyclic (**d**) cRGDK, (542.56 *m*/*z* theoretical), arrows indicate the molecular ion peak (*m*/*z*) corresponds to [M+H]^+1^. HPLC was performed using a Water Corp XBridge™ BEH C18 column (100 × 4.6 mm, 3.5 μm) using a 0–70% acetonitrile gradient using water containing 0.05% TFA as solvent A and acetonitrile containing 0.05% TFA as solvent B, at a flow rate of 1 mL/min for 8 min.

RGD is a prominent peptide motif in adhesion proteins and structural proteins, such as fibronectin, fibrinogen, collagen, and laminin. Furthermore, it acts as an anchoring site for a number of different α and β integrin-binding receptors [45]. For this study, we synthetized three linear RGD peptides (GRGD, RGDG and control RDG) and one cyclic RGD peptide (cRGDK). As shown in Figure 4, the HPLC chromatograms confirmed retention times (RT) of GRGD (3642 min), RGDG (3650 min) and cRGDK at 3600 min. Molecular masses of the purified peptides were confirmed by ESI-MS mass spectrometry, where GRGD and RGDG linear peptides were detected in the ionized state [M+1H]^+1^: 404 *m*/*z* (Figure 4b,c) and the cRGDK cyclic peptide was detected in the ionized state [M+1H]+1: 542 *m*/*z* (Figure 4d). On the other hand, control peptide RDG was detected in the ionized state [M+1H]^+1^ at 347 *m*/*z* (Figure 4a). Once peptides were characterized, SKC-CS films were treated with RGD peptides.

### 3.4. Swelling Properties of SKC-CS and Peptide Conjugation

**Figure 5:**
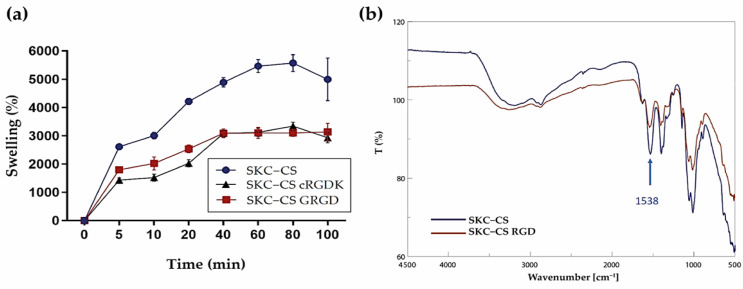
Characterization of SKC-CS films for swelling properties and presence of RGD peptide on surface. (**a**) Swelling kinetics of SKC-CS films in PBS at 37 °C; (**b**) FTIR analysis of unmodified SKC-CS film and RGD modified SKC-CS film.

Evaluation of swelling percentage in a chitosan film is important for understanding its physicochemical properties, mechanical stability, controlled release behavior, shelf life, and ensuring consistent quality in various applications [38,46]. As shown in Figure 5a, the swelling percentage of SKC-CS films reaches 2610% within 5 min, after that, the swelling percentage fluctuates around 5000% for a swelling equilibrium. In contrast, SKC-CS films functionalized with RGD peptides showed a swelling degree of 1794% and 1431% at 5 min of immersion for SKC-CS GRGD and SKC-CS cRGDK, respectively. Subsequently, for both conditions swelling percentage fluctuates around 3000% approximately.

To confirm the successful conjugation of RGD onto SKC-CS film, FTIR analysis was conducted (Figure 5). The FTIR spectra of chitosan displayed a distinctive peak at 1538 cm^−1^, indicating the involvement of the N–H bending region in the covalent conjugation of the RGD peptide using EDC and NHS. In Figure 5, a reduction in the characteristic peak of the N–H bend was observed in the FTIR spectra of SKC-CS RGD, suggesting an interaction in the primary N–H bending region.

### 3.5. Influence of RGD Peptides on Cellular Adhesion and Proliferation in SKC-CS Films

**Figure 6:**
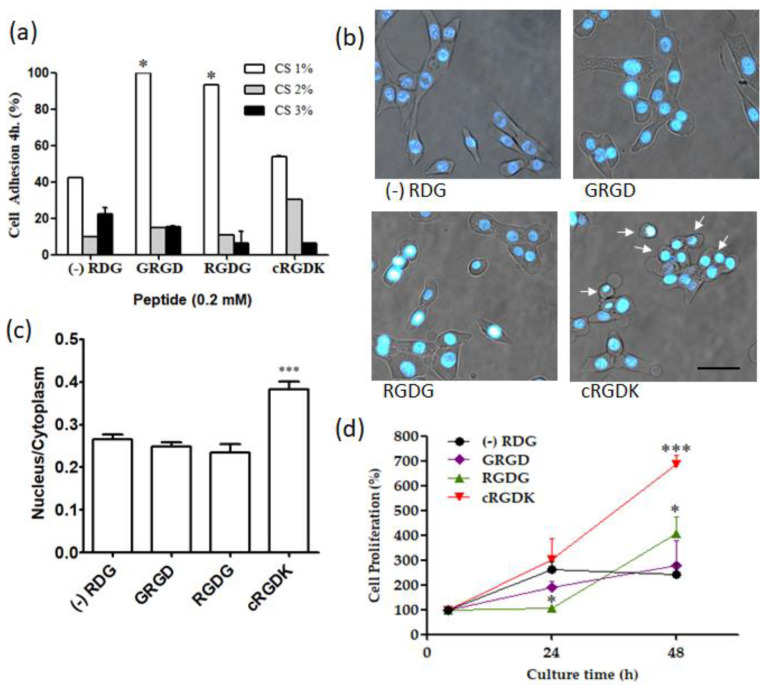
Cell adhesion and proliferation on SKC-CS films functionalized with RGD peptides. (**a**) Quantitative analysis of cell adhesion at 4 h post-culture of MEF on SKC-CS films at 1, 2 and 3% of CS concentration. Value represents cell biomass present on films with respect to total cell biomass (film plus culture plate); (**b**) Morphology of MEF cells after 4 h of seeding on SKC-CS films 1%. Nuclei were stained with Hoechst 33342. Arrows indicate rounded cells. Scale bar = 100 µm; (**c**) Ratio of nuclear to cytoplasmic area on SKC-CS films 1% after 4 h of seeding; (**d**) Quantitative analysis of cell proliferation after 24 and 48 h of cell seeding on films with RGD peptides at 1% of CS. Value represents cell biomass present on films with respect to the initial attached cell biomass (4 h; 100%). * *p* < 0.05; *** *p* < 0.01 compared to control conditions, (-)RDG.

The absence of cell adhesion motifs on CS, its highly hydrophilic nature and low protein adsorption capacity can limit cell–matrix interactions, leading to poor cellular response [47]. However, the modification of the surface of CS with adhesion peptides has been a sound approach to improve cell attachment [19,48]. Firstly, a quantitative analysis of cell adhesion was tested using resazurin after 4 h of plating. Figure 6a shows cell adhesion on SKC-CS films synthesized with 1%, 2%, and 3% of CS and functionalized with RGD peptides. Regardless of the presence of RGD peptides, films with 1% CS exhibited the highest cell adhesion. In SKC-CS (1%) films containing linear peptides, cell adhesion was twice as high compared to the control condition. On the other hand, SKC-CS (1%) films containing the cyclic peptide showed similar cell adhesion to control peptide. Figure 6b shows a photomicrograph of cells plated on SKC-CS (1%) functionalized with different RGD peptides. Under control conditions (RDG), the cells displayed a spindle-shaped morphology. However, when RGD linear peptides (GRGD and RGDG) were used, the cells appeared more flattened. In contrast, cells plated on cyclic peptide (cRGDK) exhibited a more rounded shape. A comparative analysis of the nuclear to cytoplasmic area was performed. As shown in Figure 6c, cells plated on cRGDK exhibited a higher ratio than cells in other conditions, indicating that these cells occupied a smaller area. On the other hand, Figure 6c shows the effects on cell proliferation of SKC-CS (1%) films functionalized with linear and cyclic RGD peptides. Since the cell adhesion was not similar (Figure 6a), normalization was performed by allowing the cells to attach to the films for 4 h (100% adhesion), and then the films with adhered cells were incubated for an additional 24 and 48 h. After 48 h of culture on SKC-CS films, cell proliferation using the RGDG peptide was approximately twofold higher compared to the control peptide. Remarkably, the use of the cRGDK peptide resulted in approximately threefold higher cell proliferation than the control condition.

## 4. Discussion

In recent years, the search for new sources of sustainable biomaterials from wastes of the food industry has been a strategy to mitigate the environmental impact caused by these production processes. In this sense, waste from the SKC industry is projected to be an important source of CS production in South America, especially in southern Chile. In this report, we characterized CS isolated from SKC and compared it with commercial CS to develop SKC-CS films functionalized with RGD peptides to improve cell biocompatibility. For characterization of isolated CS outcomes are consistent with CS isolation from other crab species [49,50,51]. Results showed that ash content was 2.0% for SKC-CS. In contrast, the ash content of commercial CS used in this study was 0.48%. This result shows that during the demineralization step of SKC-CS, degradation rate of biomaterial was higher in comparison to the commercial CS employed [52]. However, other studies have shown similar results in ash content for commercial CS (2.28%) [53], and other crab shells from the species *Crangon crangon* (2.5%) [54]. In this sense, high-quality grade chitosan should have less than 1% of ash content [55]. Due to the high DDA obtained from the SKC-CS (94.9%), CS was easily solubilized in an acidic solution [41,49] (Table 1).

The study of the thermal properties of biomaterials is important to determine the integrity of obtained compounds, including characteristics of use and storage [56]. DSC showed an endothermic peak mainly caused by water removal at the heating temperature [50,51] (Figure 3). In this context, the DSC thermograms are consistent with earlier studies that have suggested a correlation between higher temperature values of an endothermic peak and an increase in the water-holding capacity of chitosan [50,53,57]. Despite the CS films being synthesized with CS from two different origins (SKC and Commercial), a comparison of thermograms from films did not show differences. On the other hand, the glass transition was not visible in this experiment, probably due to the high moisture content causing that Tm to shift to a lower temperature (≈50 °C) where it appeared to merge with its glass transition [58].

On the other hand, peptides were characterized by their retention times (RT) through RP-HPLC. RT values of RGDG, GRGD and cRGDK obtained according to previous studies were values in the range RT: 3.8–3.9 [59,60]. Furthermore, the successful grafting of the RGD peptide onto SKC-CS was confirmed through FTIR spectra analysis. The obtained results provide support for previous findings indicating the conjugation of chitosan and the RGD peptide through amine groups [61,62]. However, in future studies, it will be important to quantify the presence of RGD peptides on the surface of the CS films. Additionally, investigating the stiffness conferred by the inclusion of different amounts of peptides is crucial for the potential use in different tissue engineering applications.

The chitosan film’s ability to swell significantly in PBS is due to its highly hydrophilic nature and strong affinity for salt solutions, which can be attributed to the presence of hydroxy and amino groups [63,64,65]. Moreover, the structure of chitosan films has the capacity to disrupt and restore the hydrogen-bonded network [66], this results in a chitosan film with a relatively extended chain conformation, allowing increased exposure of hydrophilic functional groups [67,68]. The restoration of this network and extended chain conformation contributes to the high swelling capacity of chitosan films. Our results showed that the functionalization of SKC-CS with RGD peptides may induce structural changes in chitosan films, affecting their swelling properties. The peptides can interact with the chitosan chains, altering the polymer conformation and the arrangement of the film matrix. These structural modifications can influence the accessibility of water molecules to the film and subsequently impact its swelling behavior [69].

Culture of cells on SKC-CS films (Figure 6a) showed that concentrations of CS higher than 1% decrease cell adhesion independently of peptide functionalization. In line with this observation, it has been reported that an inefficient capacity of chitosan (1.5%) membranes is to support fibroblasts’ adhesion and growth [70]. The effect of films with high concentrations of CS on cell behavior could be on cell membranes [71] or in cell responses to mechanical properties of the film [70]. SKC-CS (1%) films functionalized with cRGD peptide (Figure 6d) showed a significative increase in cell proliferation compared to linear RGD peptides after 48 h post-culturing. The results are consistent with previous studies, which showed a higher rate of proliferation in endothelial cells cultured for 96 h in hydrogel scaffolds containing cyclic (cRGD-PETGDA) peptides, compared to those containing linear (RGD-PEGDA) peptides [72]. Kämmerer, P.W. et al. [59], showed that titanium surfaces pre-treated with cyclic RGD peptide improves endothelial cell colonization after 3 days of cell culture in comparison to linear RGD peptide. Our findings indicate that the cyclic RGD peptide does not exhibit significant adhesion but enhances cell proliferation compared to linear RGD peptides (Figure 6). Additionally, cells plated on the cyclic RGD peptide show a more rounded shape (Figure 6b). In this regard, it is worth noting that most animal cells round up during mitosis, but the connection of cells through integrin-rich contacts is essential for cell division [73]. Therefore, the different effects observed can be attributed to the specific interaction between RGD peptides and integrin receptors on the cell surface. Linear RGD peptides lack integrin selectivity, whereas cyclization of RGD peptides improves the affinity towards integrin subtypes [74,75]. Speculatively, linear RGD peptides may activate signaling pathways that facilitate cell–substrate attachment, whereas the cyclic structure of the peptide, after attachment, could activate signaling pathways leading to increased proliferation rates. Several other advantages have been described for cyclic RGD peptides. One advantage is their prolonged and more consistent effect, which is attributed to their resistance to proteolysis [76]. Additionally, cyclic peptides have been shown to exhibit higher affinities for integrin receptors, making them advantageous for improving cell behavior [59,74,75]. Comparisons between linear and cyclic RGD peptides have demonstrated that cyclization of the peptide domain increases affinity, receptor selectivity, and enzymatic stability compared to linear peptides [74,75,77]. Moreover, cyclic RGD peptides can be used at lower concentrations due to their subnanomolar affinity for the αvβ3 receptor and low nanomolar affinity for the α5β1 receptor [78].

## 5. Conclusions

In summary, this study shows that CS isolated from SKC is an adequate source to develop films for tissue engineering purposes. Furthermore, functionalization of SKC-CS films with linear and cyclic RGD peptides improves cell adhesion and cell proliferation, respectively. Our findings are a contribution to increasing the knowledge regarding the use of new sources of chitosan to synthetize scaffolds for tissue engineering and improving the bioactivity of the surface of this material with RGD peptides.

## Figures and Tables

**Table 1:** Specification of chitosan parameters between SKC-CS and commercial CS.

Parameters (%)	SKC-CS	Commercial CS
Moisture	14.44 ± 0.04	11.67
Ash	2.0 ± 1	0.48
Nitrogen	54.7 ± 2.4	79
DDA	94.9	>75

## Data Availability

Not applicable.
